# Identification of Novel Genetic Regulatory Region for Proprotein Convertase FURIN and Interferon Gamma in T Cells

**DOI:** 10.3389/fimmu.2021.630389

**Published:** 2021-02-18

**Authors:** Zsuzsanna Ortutay, Anna Grönholm, Melina Laitinen, Melinda Keresztes-Andrei, Ismail Hermelo, Marko Pesu

**Affiliations:** ^1^Immunoregulation, Faculty of Medicine and Health Technology, Tampere University, Tampere, Finland; ^2^Faculty of Information Technology and Bionics, Pázmány Péter Catholic University, Budapest, Hungary; ^3^Computational Biology, Faculty of Medicine and Health Technology, Tampere University, Tampere, Finland; ^4^Fimlab Laboratories, Tampere, Finland

**Keywords:** Furin regulation, super-enhancer, interferon gamma, p300, proprotein convertase

## Abstract

The proprotein convertase enzyme FURIN promotes the proteolytic maturation of pro-proteins and thereby it serves as an important factor for maintaining cellular homeostasis. In T cells, FURIN is critical for maintaining the T regulatory cell dependent peripheral immune tolerance and intact T helper cell polarization. The enzymatic activity of FURIN is directly associated with its expression levels, but genetic determinants for cell-type specific *Furin* gene regulation have remained elusive. By exploring the histone acetyltransferase p300 binding patterns in T helper cells, a putative regulatory region at ca. 20kB upstream of *Furin* gene was identified. When this region was deleted with CRISPR/Cas9 the production of *Furin* mRNA was significantly reduced in activated mouse T cells. Genome-wide RNA profiling by sequencing revealed that the novel *Furin* regulator region also impacted the expression of several genes that have previously been associated with the Th1 type hall mark cytokine IFNγ regulation or function. Finally, *Furin* genetic regulatory region was found to specifically promote the secretion of IFNγ by activated T cells. In sum, our data unravels the presence of *Furin* expression regulatory region in T cells that has characteristics of a super-enhancer for Th1 cell fate.

## Introduction

The enzymes of the proprotein convertase subtilisin/kexin (PCSK) family are characterized by their common domain structure and unique cleavage target sequence (R/K)X(R/K) where X represents a 0, 2, 4, or 6 amino acid long spacer (1). PCSKs control the bodily homeostasis by regulating the bioavailability of several hormones, growth factors, other enzymes, and adhesion molecules. Proprotein convertases are also important factors in infections as they proteolytically modify various pathogenic structures, such as viral proteins, for example in HIV and SARS-Cov-2, or bacterial toxins (2). Although PCSKs show partly overlapping target specificity *in vitro*, their expression pattern and subcellular localization are divergent, and studies with knock-out animals and human mutations have unequivocally revealed distinct physiological roles for each convertase (3, 4).

The PCSK prototype enzyme, FURIN, is ubiquitously expressed and critical for embryonic development (5). In T cells, *Furin* (*Fur*) gene is upregulated by T cell receptor engagement and IL-12 cytokine and its expression is essential for maintaining intact peripheral immune tolerance, normal T helper cell polarization and activation (6–9). *Furin* is also upregulated via Toll like receptor signaling in myeloid cells wherein it exerts an anti-inflammatory function (10). Changes in *Furin* expression level are associated with a wide variety of pathological processes ranging from tumor development and progression (11), infectious diseases (12, 13) to autoimmune disorders such as rheumatoid arthritis (14) or Sjögren's syndrome (15).

The gene encoding FURIN is located on chromosome 15 in human and chromosome seven in mouse. *Furin* mRNAs arise from three different promoters, P1, P1A, and P1B leading to transcripts, which vary in 5′ untranslated region but translate into identical protein products (16). P1A and P1B promoters resemble those of housekeeping genes while the P1 promoter is inducible by cytokines [e.g., IL-12, TGFβ-1 (7, 17)] or by other external factors [e.g., thyroid hormone, hypoxia (18, 19)] that activate transcription factors including C/EBP-β, GATA-1, Smads, STAT-4 or HIF-1 (16, 19–21).

Super-enhancers or stretch enhancers (SE) are clusters of putative enhancers, binding transcriptional coactivators on an average 10-fold higher density than typical enhancers (TE) (22–24). They can be identified using the Rank Ordering of Super-enhancer (ROSE) algorithm on the basis of active enhancer marks such as histone three acetylation on lysine 27 residue (H3K27ac), mediator complex subunit 1 (MED1), and binding of lineage-specific or master transcription factors (23, 24), exceeding H3K27 acetylation level being highly efficient in super-enhancer demarcation (22, 25). Super-enhancers are cell type-specific, associating with key genes that control cell state thereby maintaining cell identity and determining cell fate (22). Trait-associated single nucleotide polymorphisms (SNPs) are consequently often enriched at super-enhancer sites of disease-relevant cells in specific diseases [e.g., in the brain for Alzheimer's disease (26)] and cancer cells often acquire super-enhancers at key oncogenes during tumor pathogenesis (22). Super-enhancers are considered as promising targets for therapeutic purposes (27).

Genetic variations linked with inflammatory conditions, such as rheumatoid arthritis or type 1 diabetes, were enriched in the T-cell super-enhancer regions (28), which underscores their clinical importance. In T cells, *Furin* expression is induced upon TCR/cytokine-mediated activation (7, 9) and in response to *Toxoplasma gondii* infection (8). However, the genetic regulatory mechanisms for *Furin* expression and their role in adaptive immunity have remained elusive. A recent study found exceedingly high histone acetyltransferase p300 binding in close genomic proximity to *Furin* gene in mouse Th1 cells (29), suggesting the presence of a super-enhancer in this cell type. In this study, we have used CRISPR/Cas9 genome-editing technology to functionally characterize this putative super-enhancer region in T cell activation and cytokine production.

## Materials and Methods

### Cell Culture and Activation

Murine thymoma cell line EL-4 was purchased from Sigma (St. Louis, MO, USA) and maintained in growth medium: RPMI-1640 (Sigma, St. Louis, MO, USA) supplemented with 10% FBS (Gibco, ThermoFisher Scientific, Waltham, MA, USA), 2 mM L-glutamine (Lonza, Basel, Switzerland), 100 U/ml penicillin (Lonza), 100 U/ml streptomycin (Lonza), 1 mM sodium pyruvate (Lonza), 4.5 g/l glucose (Lonza), 0.05 mM 2-mercaptoethanol (Sigma).

Cell clones created from EL-4 cell line were maintained in the same growth medium. Prior activation, cells were first starved in 1% FBS containing culture medium for 1 h then left unstimulated or stimulated with 10 ng/ml PMA (Sigma) and 100 ng/ml ionomycin (Sigma) for the time period mentioned. For evaluating the cytokine production from cell culture supernatants with ELISA, cells were washed once with PBS and resuspended in serum-free X-Vivo medium (Lonza) and activated as above. Thereafter cells were pelleted, and supernatants were collected and frozen until cytokine measurements with ELISA.

### sgRNAs, Plasmids, and Cloning

For designing single guide RNA (sgRNA) sequences, 150 bp sequences both up- and downstream the highest p300 binding peaks (according to GEO datasets GSM994508, GSM994516, GSM1480824) in the mouse *Furin* genomic region (chr7: 87,533,000-87,570,000 mm9) were inputed to an on-line CRISPR design tool^1^. The generated sgRNA sequences were ranked according to predicted cleavage success efficiency. Chosen gRNA sequences were further analyzed using CHOPCHOP^2^ (30). Three sgRNAs were chosen both upstream (L) and downstream (R) of the p300 binding sites either in the *Furin* promoter region (PRO) or ca. 20kb upstream *Furin* transcription starting site (putative super-enhancer site, SE) taking into account the predicted cleavage efficiency as well as possible off-target sequences. Table 1 summarizes the target sequences of chosen sgRNAs.

**Table 1:** Summary of sgRNA target sites for p300 binding area deletion.

**Name**	**Sequence**	**Genomic location (mm9)**	**Predicted cleavage efficiency**	**Off-target**
mFurin-SE-sgRNA-L1	caccGGAGGGCATGTCTTCGG CCTCCCGTACAGATAGCCcaaa	Chr7: 87,561,784-87,561,801	65.62	MM0 = 0, MM1 = 0, MM2 = 1, MM3 = 11
mFurin-SE-sgRNA-L5	caccGTGGGGGGAAGTACTCAT CACCCCCCTTCATGAGTAcaaa	Chr7: 87,561,743-87,561,760	35.83	MM0 = 0, MM1 = 1, MM2 = 6, MM3 = 116
mFurin-SE-sgRNA-L11	caccGGTGGAGGGCATGTCTAT CCACCTCCCGTACAGATAcaaa	Chr7: 87,561,781-87,561,790	45.86	MM0 = 0, MM1 = 0, MM2 = 8, MM3 = 27
mFurin-SE-sgRNA-R1	caccGGTCTTGGGATGTATCAC CCAGAACCCTACATAGTGcaaa	Chr7: 87,562,511-87,562,528	54.66	MM0 = 0, MM1 = 0, MM2 = 2, MM3 = 46
mFurin-SE-sgRNA-R3	caccgCTGTTGGTTTTGTAGGCT cGACAACCAAAACATCCGAcaaa	Chr7: 87,562,529-87,562,546	32.67	MM0 = 0, MM1 = 0, MM2 = 0, MM3 = 7
mFurin-SE-sgRNA-R12	caccgCCTATGGTCCCAGTGTTT cGGATACCAGGGTCACAAAcaaa	Chr7: 87,562,423-87,562,439	54.48	MM0 = 0, MM1 = 1, MM2 = 9, MM3 = 115
mFurin-PRO-sgRNA-L1	caccgCTGGTTCCTCCCAGATTG cGACCAAGGAGGGTCTAACcaaa	Chr7: 87,548,976-87,548,995	51.23	MM0 = 0, MM1 = 0, MM2 = 0, MM3 = 4
mFurin-PRO-sgRNA-L2	caccgTGGGGGCTTTCCCTTAGA cACCCCCGAAAGGGAATCTcaaa	Chr7: 87,548,994-87,549,011	41.95	MM0 = 0, MM1 = 0, MM2 = 0, MM3 = 10
mFurin-PRO-sgRNA-L3	caccgATGTCTGCAGTGTTTTAA cTACAACGTCACAAAATTcaaa	Chr7: 87,548,945-87,548,962	41.95	MM0 = 0, MM1 = 0, MM2 = 0, MM3 = 10
mFurin-PRO-sgRNA-R1	aaacCTCTCGAATCTGGATGGTc GAGAGCTTAGACCTACCAgccac	Chr7: 87,549,484-87,549,500	71.91	MM0 = 0, MM1 = 0, MM2 = 0, MM3 = 6
mFurin-PRO-sgRNA-R2	caccgACAGCATGTGTGTATAAA cTGTCGTACACACATATTTcaaa	Chr7: 87,549,974-87,549,990	57.24	MM0 = 0, MM1 = 0, MM2 = 2, MM3 = 43
mFurin-PRO-sgRNA-R3	caccgCAAGTGCTACCCACAATT cGTTCACGATGGGTGTTAAcaaa	Chr7: 87,550,585-87,550,602	45.35	MM0 = 0, MM1 = 0, MM2 = 0, MM3 = 8

*One hundred fifty base pair sequences from both up- and downstream of the most abundant p300 binding regions in Furin promoter area (PRO) and 20 kb upstream Furin transcription site (super-enhancer area, SE) were analyzed for targeting with Cas9 endonuclease activity. Twenty sequences of each region were evaluated according to predicted Cas9 cleavage efficiency and off-target effect, and three sequences/region were chosen for experimental procedures. Upper case letters show the sequences in mouse genome, lower case letters refer to additional nucleotides for restriction enzyme digestion during cloning to the px330 vector. Predicted cleavage efficiency and off-target prediction are assessed using ChopChop (30). Off-target values show the number of off-targets with 0 (MM0), 1 (MM1), 2 (MM2), 3 or more (MM3) mismatches*.

sgRNA sequences were cloned one by one into Cas9 expression plasmid px330 (a kind gift from Dr. Rafael Casellas, senior investigator at NIAMS, National Institutes of Health, MD, USA) following previously published protocol (31) to generate the px330-mFurin-SE-R/L and px330-mFurin-PRO-R/L constructs (**Figure 2A** shows the schematic representation of the genome editing strategy). Functionality and efficiency of these constructs were tested with T7 assay in NIH 3T3 (ATCC) cells. Briefly, genomic DNA was isolated using DNeasy kit (Qiagen, Hilden, Germany) from cells transfected with each construct individually by using TurboFect reagent (ThermoFisher Scientific, Waltham, MA, USA) according to the manufacturer's protocol. Targeted genomic area was amplified with PCR using DreamTaq DNA polymerase (ThermoFisher Scientific) and primer pairs upstream and downstream of the targeted deletion (for SE: SE-L-For: 5′-TGGTTTTCTGCCTTTGAAATGT-3′ and SE-R-Rev: 5′-CTGGGACTTCACACTCTGCT-3′; for PRO: PRO-L-For: 5′-GGGCTCAAATCCTTGCGGTA-3′ and PRO-R-Rev: 5′-CAGCTGAGCTTCCTGGACC-3′). PCR products were annealed in a reaction containing 1x NEBuffer 2 (New England Biolabs, MA, USA) and run on 10% polyacrylamide gels.

### Generation of EL-4 Cell Clones With Targeted Deletion of Putative Super-Enhancer (SE) or Promoter (PRO) Region

Stable EL-4 cell clones with deleted SE and PRO regions were generated according to a previously published protocol (32). Briefly, 24 h before transfection, EL-4 cells were adjusted to a concentration of 0.5 × 10^6^ cells/ml in complete growing medium. A total of 6 × 10^6^ cells were transfected with the mixture of 6 μg px330-mFurin-sgRNA-R and 6 μg px330-mFurin-sgRNA-L (for PRO or SE deletion) or as control 12 μg px330 vector (“empty”) constructs together with 0.6 μg pMax-GFP plasmid by using the Amaxa SF Cell Line 4D-NucleofectorTM X Kit L (Lonza). Various different pairings of sgRNA R and L constructs were used: two different pairs of PRO deleting constructs (px330-PROR1 + px330-PROL3 and px330-PROR1 + px330-PROL5) or three for the SE constructs (px330-SEL1 + px330-SER1, px330-SEL1 + px330-SER12, and px330-SEL5 + px330-SER12). The transfected cells were resuspended in complete growth medium in a concentration of 0.5 × 10^6^ cells /ml.

Seventy two hours after the transfection GFP+ cells were sorted by using FACS Aria Fusion (BD, Franklin Lakes, NJ, USA) and seeded individually (1 cell/well) into 96 well-plates (Nunc, Roskilde, Denmark). Cell clones were expanded and screened for deletion of the targeted genomic areas by PCR. For this purpose, whole genomic DNA was isolated from the cell clones using QuickExtract DNA Extraction Solution (Epicentre Biotechnologies, Madison, WI, USA) according to the manufacturer's protocol, and deletion efficiency was investigated using PCR. PCR products were run on 2% agarose gels and EL-4 cell clones showing a clear deletion at the targeted region were retained for further experiments. In case of SE region, successful deletion produced a 360 bp product instead of the 1,108 bp wild type band while PRO region deletion led to a 320 bp product and the undeleted region was 843 bp long.

### qRT-PCR Analysis of *Furin* mRNA Expression

A total of 4 × 10^6^ cells of three individual SE or PRO deleted and “empty” px330-transfected EL-4 clones were either left unstimulated or stimulated with PMA/ionomycin for 6 h. Thereafter cells were collected, washed once in PBS and RNA was extracted using RNeasy kit (Qiagen) according to the manufacturer's protocol. RNA from each cell clone was reverse transcribed using iScript Select cDNA Synthesis Kit (Bio-Rad, Hercules, CA, USA). Gene expression was investigated with real-time quantitative PCR using the CFX96 Real-Time PCR Detection System (Bio-Rad). Primer sequences used for *Furin*: F: 5′-TGAGCCATTCGTATGGCTACG-3′, R: 5′-GGACACAGCTTTTCTGGTGCA-3′ and for the housekeeping gene *RPS18*: F: 5′-GTGATCCCTGAGAAGTTCCAG-3′, R: 5′-TCGATGTCTGCTTTCCTCAAC-3′. Relative *Furin* mRNA expression was calculated according to the 2-ddCt method, statistical analysis was performed with the GraphPad Prism software applying unpaired two-tailed Student's *t*-tests, *p* < 0.05 was considered a statistically significant difference.

### Cell Growth and Viability Assays

Three individual SE or PRO deleted, or “empty” EL-4 cell clones were seeded into complete growth medium at a concentration of 3 × 10^5^ cells/ml, and the numbers of alive and dead cells were determined by staining with 0.4% Trypan Blue solution (Sigma). Additionally, cytotoxicity was investigated using the Cytotoxicity Detection LDH Kit (Roche, Basel, Switzerland) according to the manufacturer's instructions. Briefly, samples were taken from each growing cell clones daily, and split to two. Half of the samples were supplemented with 1% Triton X-100 (Sigma). One hour later supernatants were collected from Triton X-100 treated and untreated cells, and the absorbance at 490 nm (reference absorbance 680 nm) was measured. The lactate dehydrogenase activity (which is proportional to the absorbance of its colorimetric product) of the Triton X-100 treated cells was considered as 100% and the activity measured from the medium without any cells as 0%.

### RNA Sequencing and Data Analysis

Three independent SE or “empty” EL-4 cell clones were left unstimulated or stimulated with PMA/ionomycin for 6 h as described above. Total RNA was isolated using RNeasy kit (Qiagen) according to the manufacturer's protocol. RNA library (with RiboZero kit for rRNA removal) construction and RNA sequencing services (using Illumina PE150 platform) were provided by Novogene (Beijing, China). The quality of the sequenced reads was assessed using the FastQC software. Reads were aligned to the mouse genome (mm10/ GRCm38.p5) with the STAR aligner (33), and genes without transcripts in any of the conditions (“empty” or SE, unstimulated or stimulated) were filtered out of the analysis. Statistical analysis of differential gene expression was assessed by DESeq2 algorithms (34) using DESeq2 normalization based on the median ratios (35) and apeglm shrinkage (36). Experimental design for different comparisons included the genotype (SE vs. “empty”) and the status (stimulated or unstimulated) of the cells. We considered genes as differentially expressed when the Benjamini–Hochberg adjusted *p*-value [false discovery rate (FDR)] was <0.05. Clustered heatmap was generated by using the pheatmap package (Version 1.0.12.) (37) in the R software. For gene set enrichment analyses, genes were ordered on the basis of fold change values (log2FC) and gene set enrichment was analyzed using WebGestalt^3^ (38).

### Cytokine Measurements

Cytokine (IL-2, IL-4, IL-17A, TNFα, TGFβ, IFNγ) productions of three individual SE or “empty” EL-4 cell clones were assessed from PMA/ionomycin-stimulated and unstimulated cells with commercial ELISA kits (eBioscience, San Diego, CA, USA) following the protocol provided by the manufacturer. Optical densities were analyzed with the drc package (39) in R, statistical differences between sample groups were examined by comparing the log(conc) values using unpaired two-tailed Student *t*-tests, *p* < 0.05 was considered as statistically significant difference.

## Results

### p300 Binding Is Pronounced at 20 kb Upstream of *Furin* Gene in CD4+ T Helper Cells

**Figure 1:**
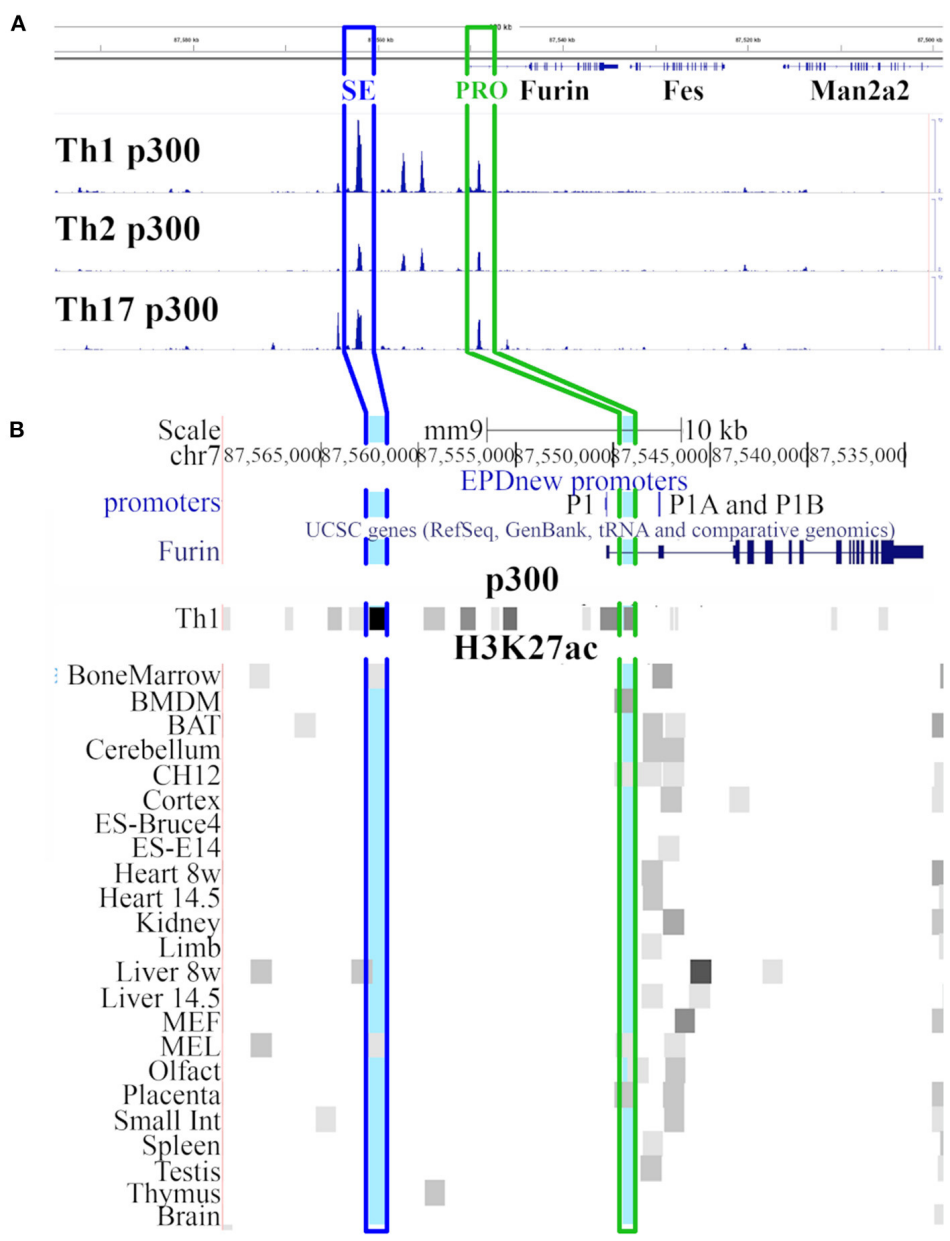
Genomic view of *Furin* region. **(A)** UCSC genome browser (41) view of region chr7: 87,500,000-87,600,000 in mouse genome (mm9 assembly) with p300 Chip-Seq data tracks from the GEO database [GSM994508 (Th1 p300), GSM994516 (Th2 p300), GSM1480824 (Th17 p300) datasets]. The heights of the p300 peaks on the Th1 p300, Th2 p300 and Th17 p300 tracks are relative to the amount of p300 bound to the chromatin. **(B)** Closer look on *Furin* gene region (modified UCSC view of region chr7: 87,533,000-87,570,000). The view shows tracks for promoters (EPD viewer hub), *Furin* gene (Refseq), p300 abundance in Th1 cells (GEO dataset GSM994508), and H3K27ac marks (ENCODE LICR data) in various tissues and cell types. Light blue marks show the genomic region with the most abundant p300 binding 20kb upstream the *Furin* transcription starting site (SE in blue frame) and within the promoter region (PRO in green frame). SE: putative super-enhancer site, PRO: *Furin* promoter site.

p300 is a histone acetyltransferase responsible for acetylating the chromatin at histone H3K27 thus activating the gene expression machinery (40). Vahedi et al. (29) sought for the key genomic regulators of CD4+ T helper cell fate by identifying putative super-enhancer (SE) regions with exceptionally high p300 loading in mouse Th1, Th2, and Th17 cells. By ranking the genomic regions according to the magnitude of p300 load they identified an area at ca. 20kb upstream of the transcription starting site of *Furin* gene that is highly occupied by p300 in Th1 type cells. This region ranked as one of the genomic sites with the most abundant p300 binding (together with *Bach2, Rgs1, Il2r, IFN*γ, *Il7r, Tbx21, Stat5* and others) and it was thus defined as a putative super-enhancer for Th1 cell fate. The evaluation of the genomic location of the p300 binding sites in different T helper cell subtypes at chr7: 87,500,000-87,600,00 of the mouse genome demonstrated the highest amount of p300 binding at putative SE site in Th1 type cells while the other investigated T cell types showed a lower amount of p300 binding (Figure 1A). In addition to the aforementioned putative super-enhancer region, high p300 binding was observed adjacent to promoter regions (PRO) of *Furin* gene in all Th subtypes. Upstream of the PRO and downstream of the SE region, two other genomic area showed enriched p300 binding in Th1 and Th2 but not in Th17 cells, whereas p300 binding was low or missing at the surrounding genomic locations.

Figure 1B shows the *Furin* region in the mouse genome (chr7:87,533,000-87,570,000; mm9 genome assembly). The two aforementioned genomic areas (SE and PRO) that showed high p300 binding in all type of T helper cells are highlighted in blue (SE) and green (PRO) color (Figures 1A,B). To further evaluate the role of p300 binding at the putative *Furin* super-enhancer site we studied the H3K27ac histone marks in several tissues from openly available data of the mouse ENCODE project (42). In tissue and cell line samples analyzed in the frame of the ENCODE project, the genomic region at the promoter area of *Furin* gene showed typically H3K27ac enrichment near *Furin's* constitutively active promoters P1A and P1B (16). Moreover, in bone marrow-derived macrophages (BMDM), erythroleukemia cell lines (CH12 and Mel), olfactory bulbs (olfact) and placenta, the H3K27ac peak at the promoter region overlapped with the p300 binding peak occurring in Th cell subgroups although this p300 peak does not overlap with any of the known *Furin* promoters but localizes between the P1 and P1A promoters. However, a H3K27ac enrichment at the putative *Furin* super-enhancer region could only be seen in bone marrow, liver and Mel samples suggesting that this genomic area is active, and in an open state only in restricted cell and tissue types.

### The Putative Super-Enhancer Regulates *Furin* mRNA Expression in Activated T Cells

**Figure 2:**
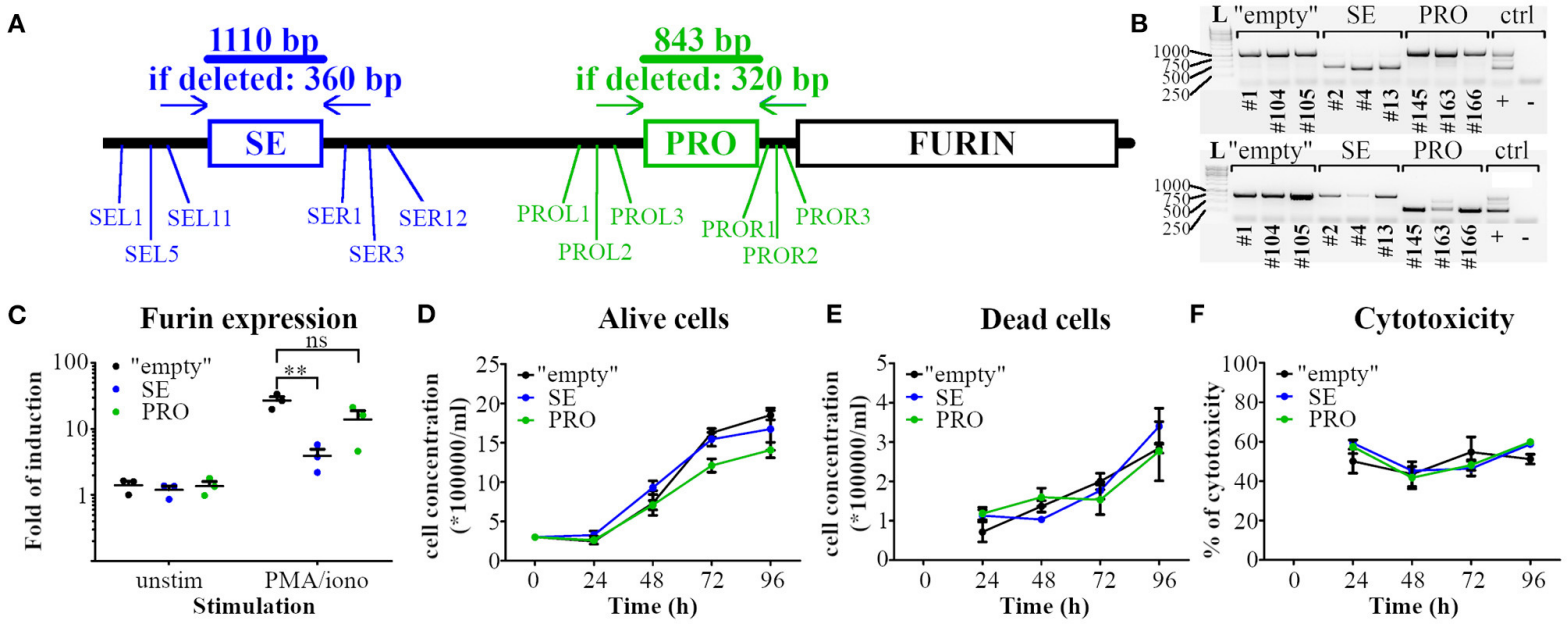
Deletion of p300 binding enrichment site upstream of *Furin* gene impairs the activation-induced *Furin* expression in T cells. **(A)** Schematic representation of the deletion strategy of the high p300-binding areas at the putative super-enhancer (SE) and the promoter region (PRO) of the *Furin* gene (FURIN). gRNAs targeting the upstream (L) and downstream (R) sequence of the p300 binding areas were designed, cloned to Cas9 expression vectors and transfected in pairs (SEL and SER or PROL and PROR) into EL-4 mouse thymoma cells. **(B)** Deletion efficiency in EL-4 cell clones was assessed with PCR using primer pairs outside of the targeted deletion area. Three independent EL-4 cell clones with full deletion of SE (above) or PRO (below) region or transfected without gRNA (“empty”) were kept for further investigations. A cell clone with heterozygous PRO region deletion was used for PCR control (control lane +). L = 1kb DNA ladder. **(C)** “Empty,” SE area deleted (SE) and PRO region deleted (PRO) EL-4 clones were left unstimulated or stimulated with PMA and ionomycin for 6 h and Furin mRNA expression was examined with qRT PCR. Normalized *Furin* expressions were compared using Student's *t*-tests (**p < 0.01). **(D–F)** Viability and proliferation of EL-4 cell clones. “Empty,” SE, and PRO clones were cultured under identical conditions, and the number of alive **(D)** and dead **(E)** cells was determined manually for 96 h. Additionally, samples from each EL-4 clone culture were taken daily and cytotoxicity was determined using LDH assay **(F)**. Data are shown as means ± SEM (*n* = 3/genotype). **(D–F)** The experiment was repeated three times with similar results).

To investigate how the identified p300 enrichment regions PRO and SE regulate *Furin* mRNA expression in T cells, we deleted these genomic areas with high p300 load in all investigated Th subtypes by employing CRISPR/Cas9 technology. To this end we cloned sgRNA sequences targeting the genomic DNA upstream (L) or downstream (R) of either the SE or the PRO area to the Cas9 expression vector px330. EL-4 mouse thymoma cell line was then transfected with pairs of either PRO or SE deleting constructs using various px330-L and px330-R combinations (Figure 2A). Transient transfection of EL-4 cells for the targeted deletions resulted in a relatively low deletion efficiency (data not shown). To overcome this challenge, we generated several stable EL-4 cell lines with deleted PRO or SE region following the guidelines provided by Bauer et al. (32). In parallel, EL-4 cells were transfected with px330 vectors without a specific sgRNA sequence to generate control cell lines (“empty”) for further experiments. The cell lines were then screened for the deletion of the PRO and SE regions and EL-4 clones with complete deletion of the targeted area (Figure 2B) were retained for further investigation.

First, we evaluated the impact of deletion of the SE or PRO area on the expression of *Furin* mRNA in resting or stimulated EL-4 clones with qRT PCR. Since this cell line shows low levels of CD3 T cell receptor on the cell surface [(43) and data not shown], we used PMA/ionomycin stimuli to bypass the T cell membrane receptor complex (44). As reported previously for primary T cells (7), T cell activation induced *Furin* mRNA expression in all three types of EL-4 cell clones (Figure 2C). *Furin* expression in unstimulated “empty,” PRO and SE EL-4 clones was similar, but when activated, the SE clones expressed significantly less *Furin* mRNA when compared to “empty” clones (Figure 2C). In contrast, the activated PRO T cell clones expressed comparable levels of *Furin* mRNA to the “empty” cells, which suggests that SE, but not PRO, p300 binding region is critical for normal activation-induced *Furin* expression in T cells.

By processing e.g. cell growth factors, FURIN is known to influence the proliferation and viability of several cell types (45–47). To evaluate if deleting the high p300-binding areas affects the cell proliferation or survival, we quantified alive (Figure 2D) and dead cells (Figure 2E) in EL-4 cell clone cultures by Trypan blue dye exclusion assay. Additionally, we investigated the cytotoxicity of the cells using the lactate dehydrogenase (LDH) leakage assay (Figure 2F). The proliferation and viability of SE and PRO clones were found to be similar to the “empty” EL-4 cell clones.

### The Deletion of the Super-Enhancer Area Does Not Affect the Splicing of *Furin* mRNA

**Figure 3:**
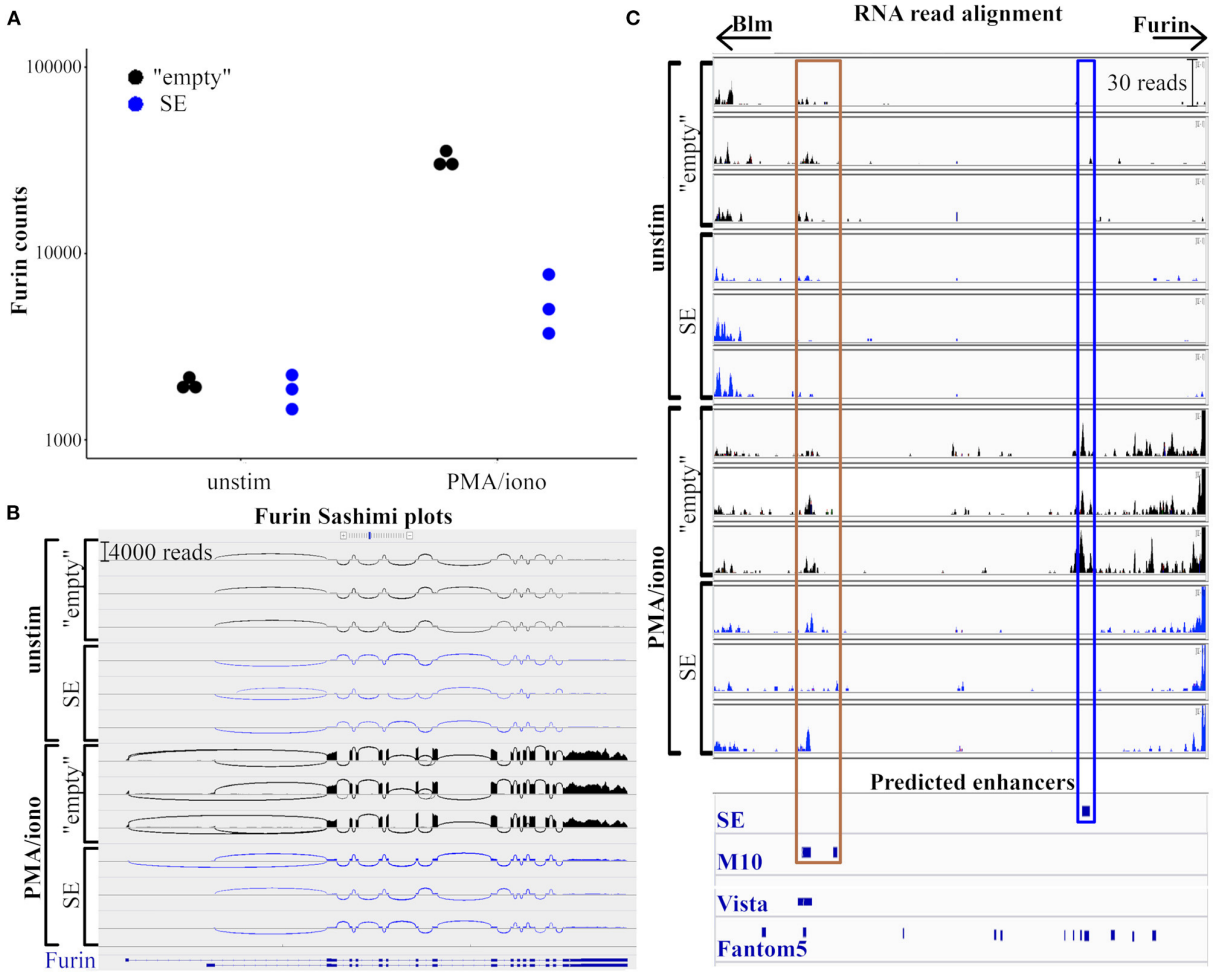
Deletion of putative *Furin* super-enhancer does not affect the RNA splicing. RNA expression in “empty” and SE EL-4 cell clones was analyzed using RNA sequencing. **(A)** Dot plot represents the counts of RNA reads aligning to *Furin* gene in unstimulated or 6 h PMA/ionomycin stimulated clones. **(B)** Sashimi plots of *Furin* region (chr7: 80,389,000-80,406,000; mm10 assembly) generated with integrative genomics viewer (IGV). The heights of the bar diagrams are proportional to read counts aligning to the given region. Loops represents reads aligning to more exons, the thickness of the line refers to the number of bridging reads. **(C)** Read coverage of the genomic region between *Furin* and *Blm* genes (chr7: 80,405,000–80,455,000; mm10 assembly). The modified IGV view shows RNA read alignments in unstimulated or PMA/ionomycin-stimulated “empty” and SE cell clones, as well as predicted enhancer sites (SE: putative super-enhancer (deleted in SE clones), M10: *Furin* enhancer (48), Vista: enhancers from Vista enhancer browser (49), Fantom5: permissive enhancers according to Fantom5 enhancer atlas (50). Brown frame marks genomic areas with validated *Furin* enhancer activity, blue frame marks the putative super-enhancer.

To further evaluate how the SE region impacts *Furin* mRNA expression, we compared the RNA transcription profiles of resting and activated SE-deleted and “empty” EL-4 T cell clones using RNA sequencing (RNAseq). Read alignment to the entire *Furin* region led to similar results as observed with qRT PCR: in steady state, both wild type “empty” and SE-deleted EL-4 clones expressed *Furin* at comparable levels. Upon PMA/ionomycin stimulation for 6 h, a 20-times increase in the number of aligned reads was observed in the “empty” clones while *Furin* expression was upregulated ca. three-fold in the SE EL-4 clones (Figure 3A).

Alternative splicing can affect the stability, localization, or translation of the mRNA, and as such, it can play an important role in gene regulation (51). There are ten alternative transcripts of mouse *Furin* in the RefSeq database^4^ (52), eight of which are predicted with computational methods while two transcripts are experimentally validated^5^. To investigate how the deletion of the putative SE region affects the exon usage of *Furin* transcripts, we generated Sashimi plots with the built-in tool of the Integrative Genomics Viewer [IGV, (53)]. Sashimi plots (54) are used to depict RNAseq reads aligning to the exons of a gene, loops representing bridges between separate exons where a read aligns to more exons thus visualizing possible alternative splicing events. We investigated the quality and the quantity of mRNA isoforms originating from the *Furin* region in wild type (“empty”) and SE-deleted cell clones (Figure 3B). In steady-state, we could not detect any marked differences in *Furin* transcription between the “empty” and SE EL-4 clones; the quantity of the reads aligning to any given genomic location (the heights of the bars on the diagrams) were similar, and the junction coverage, the number of reads aligning to separate exons (represented by the thickness of the loops on Figure 3B), was also equal. The activation of T cells for 6 h resulted in a pronounced elevation (ca. 15 to 20 times) in all read counts aligning to the translating exons of *Furin* in the “empty” cell clones whereas the induction of *Furin* expression in SE-deleted cell clones was reduced globally (ca. three times more aligning reads across all encoding exons).

Gene expression can also be regulated by lncRNAs, especially those transcribing from the antisense strand of the protein-coding DNA (55). We observed the expression of a known lncRNA gene (*Gm44851*) overlapping the alternative first non-coding exon of *Furin*, with a low transcription level in steady-state (two to six reads both in “empty” and SE EL-4 clones). PMA/ionomycin stimulus led to a ca. 100-fold elevation in *Gm44851* transcription, however, there was no statistically significant difference between the “empty” and SE-deleted EL-4 clones (data not shown).

The transcriptional activity at the active enhancers results in the production of enhancer RNAs (eRNAs), which may regulate their target gene by altering the chromatin architecture and histone modification, or by recruiting specific proteins to the enhancers (56). Super-enhancers are reported to produce higher amounts of eRNAs than typical enhancers (22). To evaluate eRNA transcription at the putative *Furin* super-enhancer region we compared the RNA alignments of resting and PMA/ionomycin stimulated “empty” and SE-deleted cell clones. We also examined the read alignment to the M10 area, a genomic region (30 kb upstream *Furin*, 10 kb downstream *Blm*) with reported *Furin* enhancer activity (48) (Figure 3C). In “empty” EL-4 clones, T cell activation resulted in 20–30 eRNA reads aligning to the super-enhancer region indicative of activation-induced eRNA production. Logically, in resting or activated SE clones where the p300-binding region is deleted, no eRNA reads aligned to the super-enhancer region. In contrast, the eRNA production at M10 in EL-4 cells was not evidently dependent of the SE region or T cell activation (8–16 aligning eRNA reads/condition).

### The *Furin* Super-Enhancer Region Regulates Gamma Interferon Gene Signature

**Figure 4:**
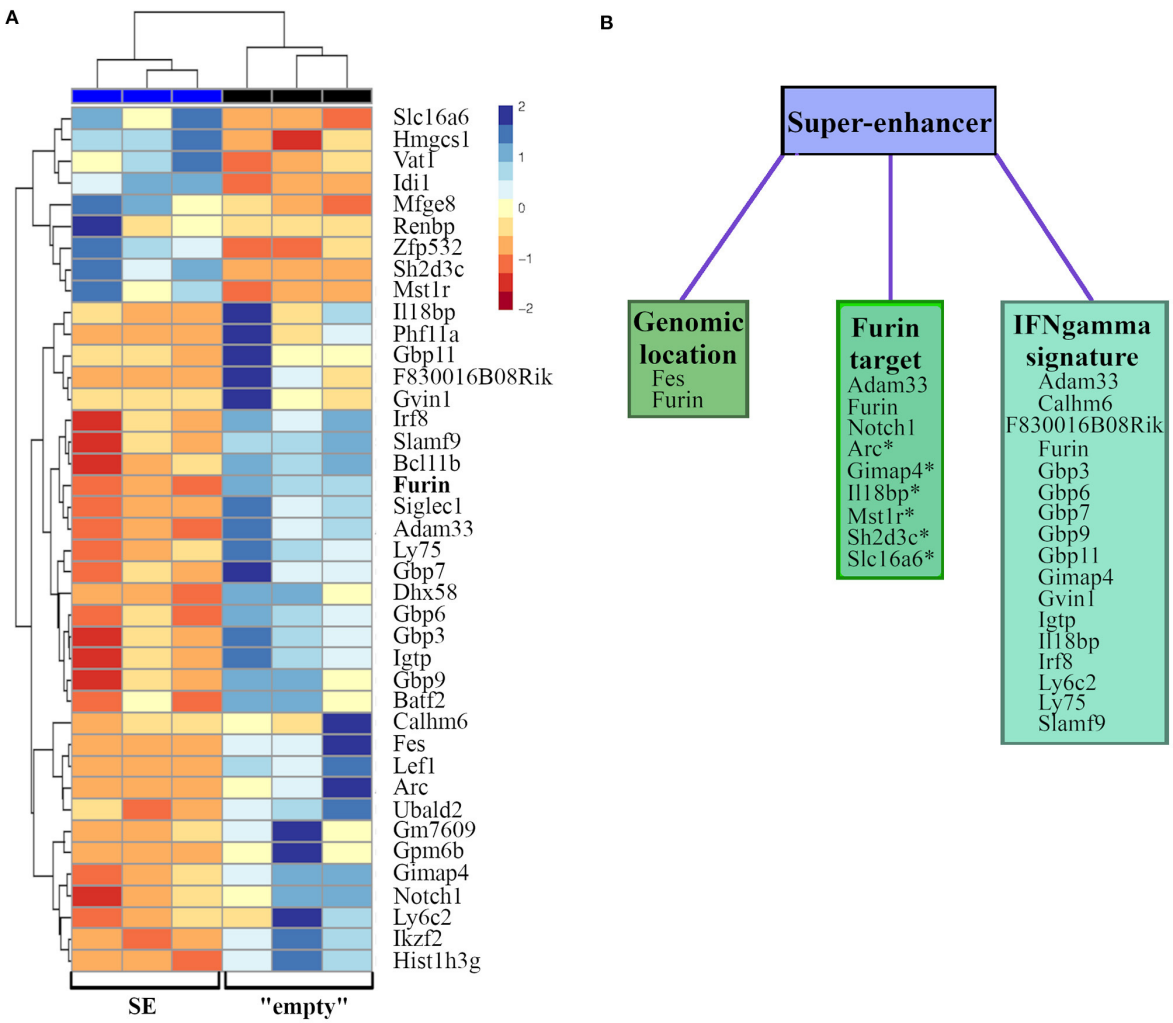
*FURIN* SE regulated RNA expressions in activated EL-4 T cells clones. “Empty” and SE EL-4 T cell clones were stimulated for 6 h with PMA and ionomycin (*n* = 3/genotype). Gene expression of clones was analyzed using RNA sequencing. **(A)** Heatmap representation of significantly differentially expressed genes (*p* adj < 0.05). Genes are arranged according to the expression pattern similarity, colors representing the log2-transformed internally normalized RNA read counts. **(B)** Differentially expressed genes are categorized into groups based on common features, such as close genomic location, validated or predicted (*) FURIN cleavage, or tie to IFNγ.

Next, we compared the genome-wide RNA expression profiles of unstimulated and stimulated (6 h, PMA/ ionomycin) “empty” and SE-deleted EL-4 cell clones (Supplementary Figure 1, Supplementary Table 1). This analysis revealed differential expression of a total of 141 genes, among those only immune system process (GO:0002376) associated genes were significantly over-represented (FDR = 0.04, data not shown). As the impact of SE on the *Furin* expression was only significant in activated cells, we next compared directly the gene expression profiles of activated “empty” and SE clones (Figure 4A). This analysis revealed that the *Furin* SE region significantly regulated the expression of a total of 40 transcripts (p adj < 0.05), some of which have been reported to share common ontologies or other features (Figure 4, Table 2). For example, SE region impacted the expression of both *Furin* and *Fes*, the gene directly downstream and in a short genomic distance to *Furin*. Two genes encoding previously identified FURIN target molecules [NOTCH1 (62) and ADAM33 (57)] were among the SE-dependent genes as well. Additionally, FURIN cleavage could be predicted with the ProP software^6^ (77) in the protein product of six other SE-dependent genes, namely *Arc, Gimap4, Il18bp, Mst1r, Sh2d3c*, and *Slc16a6* genes (Table 2).

**Table 2:** Groups of differentially expressed genes.

**Genomic location**		**Furin target**		**IFNγ** **signature**	
**Gene name**	**Genomic location (mm9)**	**Gene name**	**References/target sequence (probability)**	**Gene name**	**References**
Furin	Chr7: 87,534,080-87,547,648	Adam33	(57)	Adam33	(58)
Fes	Chr7: 87,522,644-87,532,832	Furin	(59)	Calhm6	(60, 61)
		Notch1	(62)	Furin	(7)
		Arc	DSQRWKK|SI (0.650)	F830016B08Rik	(63)
		Gimap4	EKARIRR|EY (0.645)	Gbp3, Gbp6, Gbp7, Gbp9, Gbp11	(64, 65)
		Il18bp	HTSREHR|NT (0.529)	Gimap4	(66)
		Mst1r	APKRRRR|GA (0.703)	Gvin1	(67)
		Sh2d3c	YHGRIPR|EV (0.518)	Igtp	(68)
		Slc16a6	GPQRRRR|GW (0,615)	Il18bp	(69, 70)
				Irf8	(71)
				Ly6c2	(72–74)
				Ly75	(75)
				Slamf9	(76)

*Wild type “empty” and super-enhancer deleted SE EL-4 clones were stimulated (PMA/ionomycin, 6 h). Total RNA was isolated and submitted to RNA sequencing. Forty differentially expressed genes were found with the DESeq2 algorithms (34), which could be grouped to three categories based on similarities in genomic localization, holding FURIN target sequence or their common relation to IFNγ*.

A remarkable number of the SE-dependent genes have previously been associated with the Th1 signature cytokine IFNγ. Many of these exert GTPase activity [*F830016B08Rik* coding immunity-related GTPase 4 (IFGGA4) protein], or are IFNγ-inducible guanylate binding proteins (*Gbp3, Gbp6, Gbp7, Gbp9, Gbp11*), *Gimap4, Gvin1* (also known as *Vlig1*) and *Igtp*). Also several genes that either regulate or are regulated by IFNγ were found to be SE-dependent. *Adam33* is regulated in an IFNγ dependent manner in airway smooth muscle cells of asthma patients (58), *Calhm6* (also known as *Fam26f* or *Inam*) both regulates IFNγ in NK cells (60) and is itself upregulated by IFNγ in lymphocytes (61), *Il18bp* is regulated by IFNγ (70) and it inhibits the levels of circulating IL-18 with a consequential IFNγ reduction (69). *Irf8* is an IFNγ inducible transcription factor with a crucial role in myelopoiesis (71). *Ly6c* expression positively correlates with IFNγ production by effector T cells (72, 73), and endogenous IFNγ induces *Ly6c* expression (74). *Ly75* (*Dec205/CD205*) is a surface molecule on a subset of dendritic cells (75). *Slamf9* expression is also upregulated upon IFNγ stimulus in bone marrow-derived macrophages (76). Lastly, also *Furin* has been shown to control *ifn*γ expression, and both *Furin* and *IFN*γ expression are co-regulated via the IL-12/STAT4 pathway (7). As the number of significantly differentially expressed genes in actvated “empty” and SE clones was limited, we further ranked all genes according to the difference in expression levels between stimulated SE and “empty” cells (log2foldchange) and evaluated enriched gene ontology terms and KEGG pathways. These analyses did not reveal any additional gene set enrichments between the activated “empty” and SE clones (data not shown).

In sum, RNAseq data indicate that the *Furin* SE region regulates the transcription of genes that are located at its vicinity (*Furin, Fes*), FURIN target molecules as well as several genes that are associated with the IFNγ cytokine regulation or function.

### *Furin* Super-Enhancer Promotes the Production of Th1 Cytokine IFNγ in Activated T Cells

**Figure 5:**
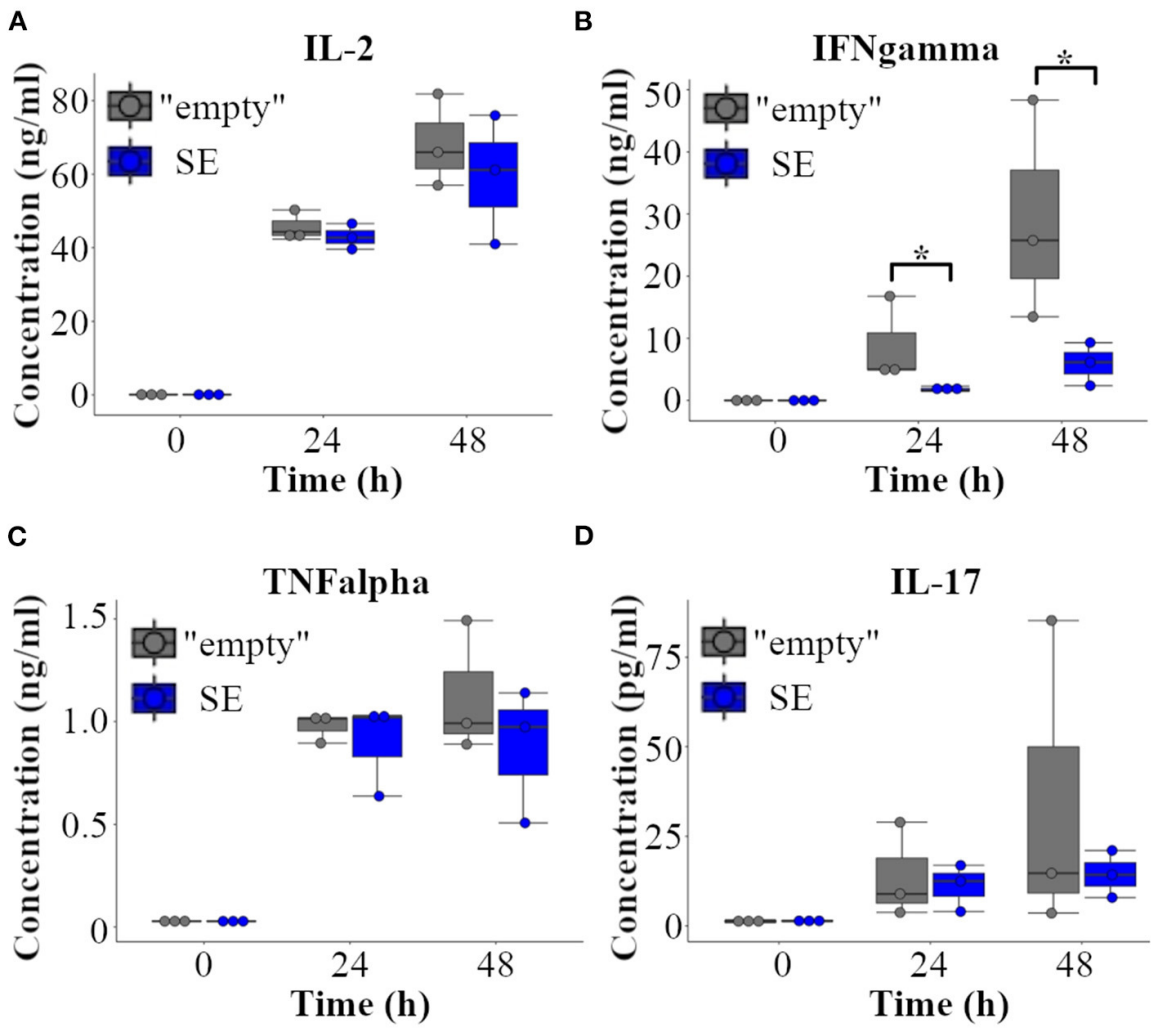
*Furin* super-enhancer regulates IFNγ in activated T cells. “Empty” and SE-deleted EL-4 T cell clones (*n* = 3/genotype) were left unstimulated or activated with PMA and ionomycin for 24 and 48 h. The production of **(A)** IL-2, **(B)** IFNγ, **(C)** TNFα and **(D)** IL-17 were evaluated from the supernatants with ELISA. The cytokine levels were compared using Student's *t*-test (*p < 0.05). Three independent “empty” and SE EL-4 clones were stimulated three times, ELISA measurements were repeated two to three times leading to similar results.

Data from the RNA sequencing experiments imply that *Furin* SE may have a role in regulating Th1 type responses, such as the production of IFNγ. To test this hypothesis directly, we evaluated the production of T cell cytokines in “empty” and SE EL-4 T cell clones. To this end, EL-4 cell clones were stimulated with PMA/ionomycin for 24 h and 48 h, or left untreated, and cytokine levels were measured in the cell culture supernatans using commercial ELISA kits. Remarkably, deleting the *Furin* SE region significantly reduced the production of IFNγ by activated T cells at both 24 and 48 h (Figure 5) whereas the production of IL-2, TNFα, and IL-17 were unaffected by SE deletion. Th2 type hallmark cytokines were below the level of detection in both “empty” and SE EL-4 cell clones at all tested time points (data not shown). These data indicate that the p300 binding enrichment site ca. 20 kb upstream of *Furin* gene has a potential to serve as a genuine super-enhancer region for IFNγ production and T helper 1 type cell fate.

## Discussion

Although the structure of *Furin* gene and its promoters have been described already in the 1990's, its genetic regulation is still not well-understood. The development of DNA sequencing technologies now allows for further deciphering regulatory sequences that control the spatial and temporal expression of genes. ChIP-seq using antibody against the acetyltransferase and transcriptional coactivator p300 protein predicts the presence of active enhancers with high accuracy, more p300 binding referring to higher transcriptional activity of a target gene (40). p300 has a dual role in the regulation of gene expression: as a chromatin-modification enzyme it can regulate chromatin accessibility, and by recruiting transcription factors it can help in the assembling of the transcriptional machinery. Further, genes determining cell identity are associated with super-enhancers, which are marked by exceptionally high p300 binding. This feature is useful for identifying genes that play key roles in defining cell fate. The exceptionally high p300 histone acetylase binding upstream of *Furin* gene in mouse Th1 cells (29) referring to the presence of a super-enhancer (SE) at this genomic area appoints *Furin* as a critical element of Th1 fate. Indeed, FURIN has previously been reported to regulate IFNγ (7) and to be essential for generating pathogen-specific Th1 lymphocytes after *Toxoplasma gondii* infection (8). However, a lower level of p300 binding was also found in the promoter region of other investigated T helper cell types (Th1, Th2, and Th17) as well as in the SE region of Th2 and Th17 cells. The quantity of p300 binding can be decisive in defining the rate of RNA transcription, and two other binding sites with lower p300 load were found upstream of the PRO and downstream of the SE area in Th1 and Th2 cells. Understanding the cell-specific regulation of *Furin* expression would benefit from the functional evaluation of the other two putative enhancer sites in the future.

In this work, we examined the regulatory role of the genomic regions appointed by high levels of p300 binding in mouse Th cells by generating cell clones with complete deletion of these sequences in *Furin* promoter region (EL-4 PRO) or ca. 20 kb upstream *Furin* (EL-4 SE) and comparing *Furin* expression to that of the wild type (EL-4 “empty”) cells. Surprisingly, deletion of the p300 binding sequence in the promoter region has not caused any changes of *Furin* expression in either steady-state or following stimulation although analysis of this sequence revealed putative binding sites for different transcription factors (such as EVI-1, HAND-1, PAX4, and c-REL, data not shown). Notably, the p300 peak present in the PRO region in primary mouse T cells does not overlap with the reported *Furin* promoter sequences but it is rather situated between the inducible P1 and the housekeeping-like P1A and P1B promoters.

On the other hand, deletion of the SE region led to significant reduction in stimulation-dependent upregulation of *Furin* expression while, in steady-state, *Furin* expression levels were similarly low in all three types of EL-4 cell clones. The importance of the SE region in EL-4 cells was underlined by the fact that this genomic region could be deleted referring to an open—thus accessible—chromatin state in this cell type. In contrast, targeting the same sequence in NIH 3T3 cells proved to be unsuccessful (data not shown) suggesting that in fibroblast cells the SE area can be closed, and as such, not accessible for the activity of Cas9 endonuclease. In line with the ubiquitous expression of *Furin*, the PRO region could be deleted in both EL-4 and NIH 3T3 cells (data not shown). The deletion of the SE sequence caused a significant decrease but not complete lack of *Furin* mRNA in stimulated cells, allowing the analysis of cellular processes which are dependent on dose-dependent *Furin* expression. For example, although *Furin* deficiency in mouse T cells was associated with increased T cell numbers (6), decrease in FURIN level seems not to influence cell proliferation and survival. However, it is important to note that the EL-4 cell line model might not fully reflect the true role of the SE region and it will be thus important to replicate our findings in more physiological settings, ideally by generating an SE targeted mouse strain.

While in T cells complete lack of FURIN results in differential expressions of several genes (9), the deletion of the putative SE area affected only a restricted set of genes. These included genes that are in close genomic distance to the putative SE, reported and predicted FURIN targets, and IFNγ signature genes. The regulatory effect of SE on *Fes* can be explained by the close genomic distance of *Furin* and *Fes* rendering these two genes to the same genomic regulatory domain. However, *Blm*, upstream of *Furin*, and in a similar (shorter) distance from the SE region as *Fes* (ca. 26 vs. 29 kb), was not differently expressed in stimulated SE and “empty” EL-4 clones although enhancers are able to activate gene expression independently of their orientation (78). The fact that FURIN target proteins are among the differentially expressed genes is in keeping with the observation that the expression of FURIN and other proprotein convertase enzyme genes are often co-regulated with their cognate target molecules (79).

The putative super-enhancer influenced the expression of several genes, which are known to be regulators of or being regulated by IFNγ. ELISA experiments demonstrated that IFNγ was produced at lower levels by SE-deleted EL-4 clones compared to the wild type “empty” EL-4 cells. This finding is in accordance with the presence of the investigated super-enhancer in Th1 cells: super-enhancers are thought to regulate genes which are decisive for cell identity. Interestingly, complete deletion of *Furin* in T cells seems to have wider effects on cytokine production than SE-deletion in EL-4 cells. For example, while *Furin* KO T cells produce elevated levels of IL-2 in steady-state but have impaired IL-2 responses upon T cell stimulus (9), SE-dependent reduction in *Furin* expression does not influence IL-2 production in EL-4 cells indicating a dose-dependent role for *Furin* in IL-2 regulation. TNFα and IL-17 productions remained unaffected by FURIN levels which is in accordance with earlier findings in FURIN cKO mice (6). Collectively, these data indicate that *Furin* may have an expression level dependent mode of action on T cell proliferation, activation and T helper cell polarization

To understand the mechanism by which the SE region regulates *Furin* expression, we investigated the possibility of gene regulation via alternative RNA splicing. T cell activation by PMA/ionomycin stimulation caused more RNA fragments aligning to *Furin* gene. Similarly to this finding, phorbol esters were reported to enhance *Furin* P1 promoter activity in neuronal cells (80). However, *Furin* mRNA splicing profiles were intact in SE-deleted EL-4 clones, which suggests that RNA splicing is independent of the SE region. In addition, we could not detect significant difference in the transcription of the lncRNA gene *Gm44851* either in steady-state or following stimulation. We also noticed eRNA transcription at the putative super-enhancer site in “empty” EL-4 clones proving the enhancer activity of this genomic region. Upon stimulation, the amount of RNA reads aligning to this area increased suggesting elevated enhancer activity.

However, deletion of the SE sequence or PMA/ionomycin stimulation did not have a clear effect on the transcription of the earlier reported *Furin* enhancer, M10. The SE region includes target sequences for a different set of transcription factors (e.g., OCT1, PAX-6, NKX2-5, and HNF-1). The regulatory role of these transcription factors on *Furin* expression or in Th cell fate remains yet to be established. Notably, the SE region also includes a putative binding site for T-bet (GSM949921), a key regulator of Th1 polarization. Deletion of SE region can thus potentially disturb Th polarization (81) also by diminishing the T-bet dependent Th1 polarization events.

Our data demonstrate that the predicted super-enhancer indeed regulates *Furin* expression in stimulated mouse T cells. There are also reported super-enhancers upstream *Furin* gene in human cells, cell lines (CD4+CD25-Il17+ Th17, K562, Ly4, HeLa) and organs (liver, tonsils, small intestine, pancreatic islets and adipose nuclei) according to the super-enhancer database, dbSuper (82). Single nucleotide polymorphisms (SNPs) in the reported genomic regions are associated with stroke [rs4932370 (83)], blood pressure variation [rs4932371 (84), rs8029440 (85)], and asthma [rs8029440 (86), rs74874915 (87)]. Super-enhancers are emerging targets of therapies where cell-type specific gene regulation is impaired, such as in cancers (23, 88) or autoimmune diseases (28, 89, 90). Targeting the super-enhancer would enable to influence *Furin* expression only in selected conditions when the super-enhancer is in an active state.

FURIN has recently emerged as a potential target in infectious diseases and cancer. Especially, as the critical determinant of SARS-CoV-2 infectivity—the spike protein, is now known to be processed by FURIN, inhibitors may have clinical value in harnessing this global epidemy (91). Upregulated FURIN level/ activity can have both beneficial and pathological effects in malignant diseases. FURIN level is elevated in various tumor cell lines and primary tumors (11), and FURIN activity promotes many cancer-related processes, such as cell proliferation, migration or vascularization, for example (11). It is thus not surprising that FURIN inhibitors are suggested in the treatment of various cancers and to prevent infections (92). On the other hand, *Furin* overexpression is not a common feature of diseases associated with *Furin*: for example, *Furin* is downregulated in prostate cancer (93) and *Furin* mRNA and protein levels are significantly decreased in the brain of Alzheimer's disease patients (94). Additionally, in hepatocellular carcinoma, *Furin* overexpression associates with better prognosis (95).

In sum, our understanding on the regulation of *Furin* and its importance remains still incomplete. However, it will be critical to continue work on the subject as better understanding of this matter may potentially have clinical value in understanding the pathogenesis and treatment strategies for a wide range of detrimental diseases.

## Data Availability Statement

The datasets presented in this study can be found in online repositories. The names of the repository/repositories and accession number(s) can be found below: https://www.ncbi.nlm.nih.gov/geo/, GSE158456.

## Author Contributions

ZO organized experiments and participated in generating EL-4 clones, carried out and analyzed RNA sequencing, carried out ELISA, and drafted the manuscript. AG and ML designed and cloned gRNAs. MK-A transfected EL-4 cells and carried out cell viability assays, determined alive/dead cell counts, carried out qRT PCR. IH generated EL-4 cell clones. MP supervised research and reviewed data and revised the paper. All authors contributed to the article and approved the submitted version.

## Conflict of Interest

MP was affiliated with Fimlab ltd without employment. The remaining authors declare that the research was conducted in the absence of any commercial or financial relationships that could be construed as a potential conflict of interest.

## Footnotes

^1^http://crispr.mit.edu

^2^http://chopchop.cbu.uib.no/

^3^http://www.webgestalt.org/

^4^https://www.ncbi.nlm.nih.gov/refseq/

^5^https://www.ncbi.nlm.nih.gov/nuccore/NM_011046.3,NM_001081454.2,XM_011250818.2,XM_011250819.3,XM_011250820.3,XM_011250821.2,XM_011250822.2,XM_011250823.1,XM_030242209.1,XM_030242210.1

^6^https://services.healthtech.dtu.dk/service.php?ProP-1.0
