# Supplementary material for: Identification of Novel Genetic Regulatory Region for Proprotein Convertase FURIN and Interferon Gamma in T Cells
Source: Front Immunol. 2021 Feb 18;12:630389. doi: 10.3389/fimmu.2021.630389 (PMC7930619; doi:10.3389/fimmu.2021.630389)

Supplementary Figure 1. A heatmap of differentially expressed genes in “empty” and SE EL-4 T cell clones.

“Empty” and SE EL-4 cell clones (3+3) were left unstimulated or stimulated with PMA and ionomycin for 6 h and genome-wide transcription profile was assessed with RNA sequencing. Heatmap depicts the 141 differentially expressed genes (p adj <0.05) in “empty” versus SE clones in steady-state or following stimulation.

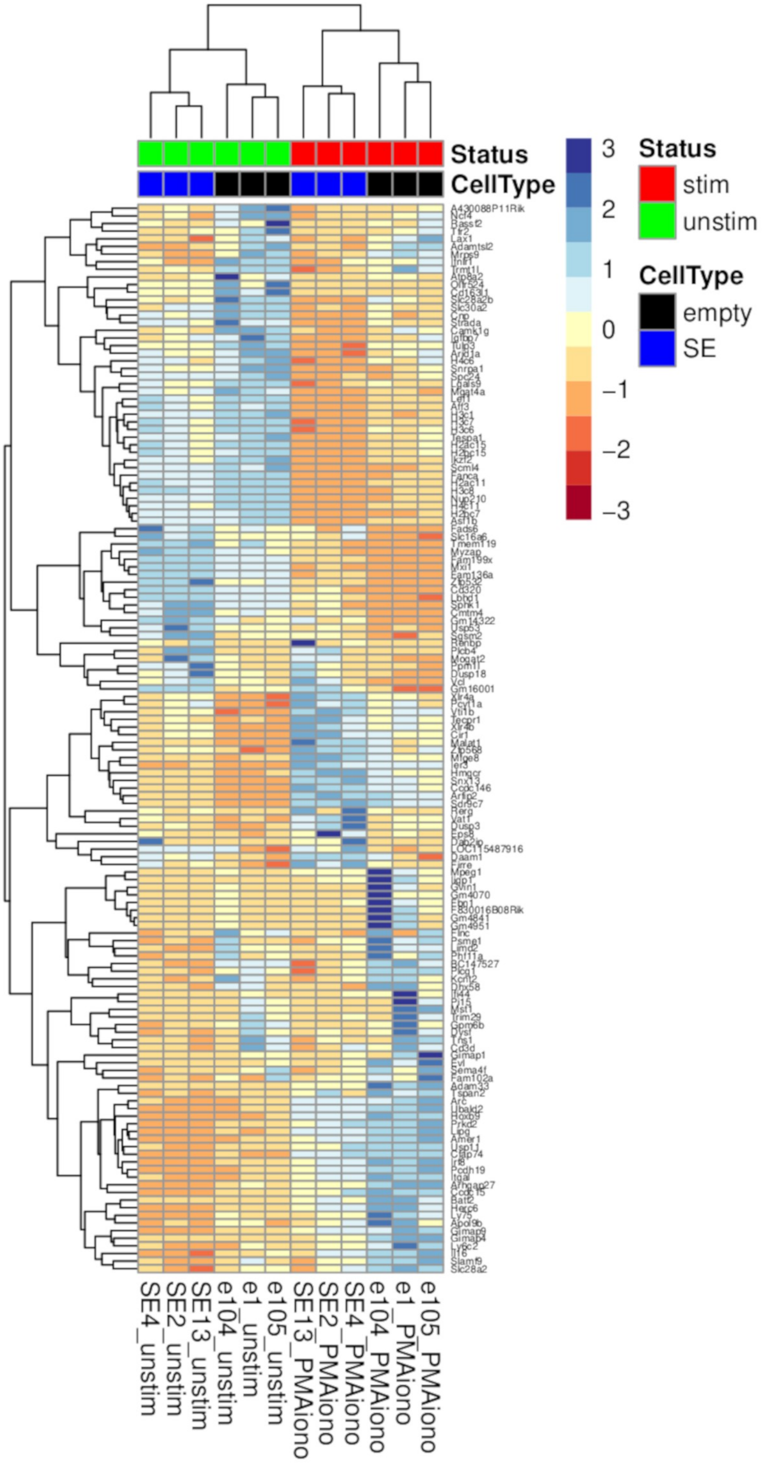

Supplement: Supplementary file 2 [file Data_Sheet_2.PDF]
